# Gill infection in *Macrodon ancylodon* and *Nebris microps* caused by *Lernanthropus* sp. (Copepoda: Lernantropidae) in the Brazilian Amazon

**DOI:** 10.1590/S1984-29612025029

**Published:** 2025-06-13

**Authors:** Alessandra Epifanio Rodrigues, Gilmar da Silva Pantoja, Vanessa Mayara Souza Pamplona, José Ledamir Sindeaux, Michele Velasco

**Affiliations:** 1 Programa de Pós-graduação em Saúde e Produção Animal na Amazônia – PPGSPAA, Universidade Federal Rural da Amazônia – UFRA, Belém, PA, Brasil; 2 Instituto da Saúde e Produção Animal, Universidade Federal Rural da Amazônia – UFRA, Belém, PA, Brasil; 3 Laboratório de Integração Morfo-molecular e Tecnologias – LIMT, Universidade Federal Rural da Amazônia – UFRA, Belém, PA, Brasil; 4 Universidade da Amazônia – UNAMA, Belém, PA, Brasil; 5 Programa de Pós-graduação em Reprodução Animal – REPROAMAZON, Universidade Federal Rural da Amazônia – UFRA, Belém, PA, Brasil

**Keywords:** King weakfish, Smalleye croaker, Fish parasites, Copepods, Amazon, Pescada-gó, Pescada-banana, Ictioparasitos, Copédodes, Amazônia

## Abstract

The research reports the occurrence and morphologically describes *Lernanthropus* sp. in the gills of sciaenid fish *Macrodon ancylodon* and *Nebris microps*. Specimens of the species were collected and examined. Ectoparasitic copepods with morphological characteristics inherent to the genus *Lernanthropus* were observed on the gills of the hosts and exhibited the same parasitic prevalence (25%). The copepod in *M. ancylodon* was male and did not exhibit a sac-like projection with eggs, with poorly developed antennae in the anterior region, a body measuring 4.33 mm in length, and a head distinct from the trunk measuring 0.96 mm (length) x 1.15 mm (width). While in *N. micros*, it was female, with a very evident sacuolar projection, with more developed and larger antennae than the specimen obtained in *M. ancylodon*, the anterior part of the trunk (2.1 mm) narrower than the posterior (3.2 mm) and a continuous plate to the trunk in an oval circular shape, with a sacuolar projection (4 mm). It should be noted that until now, there has been no report of the occurrence of this genus in the hosts studied in the state of Pará, which makes this work an important record for the knowledge of the ichthyosanitary status in this region.

## Resumo

A pesquisa relata a ocorrência e descreve morfologicamente *Lernanthropus* sp. em brânquias de peixes cienídeos *Macrodon ancylodon* e *Nebris microps*. Foram coletados e examinados exemplares dessas espécies. Observou-se nas branquiais o ectoparasito copépode com características morfológicas inerentes ao gênero *Lernanthropus* nos ho spedeiros e apresentaram a mesma prevalência parasitária (25%). O copépode em *M. ancylodon* era macho e não apresentava projeção sacuolar com ovos, antenas pouco desenvolvidas na região anterior, corpo com 4,33 mm de comprimento e com cabeça destacada do tronco, com 0,96 mm (de comprimento) x 1,15 mm (de largura). Enquanto em *N. micros*, era fêmea, com projeção sacuolar bem evidente, com antenas mais desenvolvidas e maior que o exemplar obtido em *M. ancylodon*, a parte anterior do tronco (2,1 mm) mais estreita que a posterior (3,2 mm) e a placa contínua ao tronco de formato circular ovalado, com projeção sacuolar (4 mm). Ressalta-se que até o presente momento, não existe relato da ocorrência desse gênero nos hospedeiros estudados no estado do Pará, o que torna este trabalho um importante registro para o conhecimento do estado ictiosanitário nessa região.

## Introduction

Fishing plays a crucial economic role in the state of Pará, Brazil, wherein the household per capita fish consumption in 2018 was four-fold higher than the national average, reaching 11.1 kg/inhabitant compared with 2.8 kg/inhabitant in Brazil (FAPESPA, 2023).

Among the most commercially important fish species in the state are members of the Sciaenidae family, commonly known as weakfish or croakers. Sciaenid species are widely distributed and abundant along the Brazilian continental shelf (Condini et al., 2022). Owing to their commercially attractive size, many species are targeted by fisheries and represent a significant portion of fishing landings (Chaves & Robert, 2003; Vianna & Almeida, 2005; Chao et al., 2015).

Among the most commercially important species are *Macrodon ancylodon* (Bloch & Schneider, 1801) and *Nebris microps* (Cuvier 1830), both of which are significant in national and regional markets. *M. ancylodon* (king weakfish) is widely distributed along the South American Atlantic coast (Ikeda, 2003; Santos et al., 2003). It is a species of demersal, marine fish, belonging to the Sciaenidae family, also found in estuarine waters, their diet primarily comprises shrimp, small fish, and squid (Ikeda, 2003; Santana et al., 2019).

*N. microps* (smalleye croaker) is a demersal species widely distributed along the American coast from northern to southeastern Brazil (Paiva & Cergole, 1988). Although it is frequently caught incidentally by shrimp trawlers in Brazil, there is little information available on its life history. Reproductive activity is intense in summer, with spawning occurring throughout the year, and individuals reaching adulthood at around two years of age (Giombelli-da-Silva, 2023).

In their natural habitat, these fishes face various parasitic challenges, particularly ectoparasites, such as copepods, whose infestations can impair the development, health, and appearance of commercially valuable fish species, hindering their marketability (Fonsêca et al., 2000). Copepods represent the largest and most diverse group of crustaceans, with most species being wild and playing crucial ecological roles as vital components of the aquatic food web and ecosystem maintenance, whereas others are parasitic, primarily affecting fish populations (Huys & Boxshall, 1991).

The genus *Lernanthropus* (Copepoda: Lernanthropidae) is known to cause pathological effects in its host, and the lesions caused by these parasites can serve as entry points for secondary microbial infections, which promotes the development and transmission of infectious diseases (Fonsêca et al., 2000).

Although the genus *Lernanthropus* has not been described in the Amazon region, this genus warrants attention as it comprises over 110 species worldwide, but only 35 are parasites of marine teleostous fishes, and is the most prevalent genus among parasitic copepods of fish, it is also the most widespread genus in the Lernanthropidae family, with documented presence in the Atlantic, Indian, and Pacific oceans (Luque & Farfán, 1990; Cavalcanti et al., 2013; Taborda et al., 2014).

This study aims to present the occurrence and provide a morphological description of *Lernanthropus* sp. in king weakfish and smalleye croaker obtained from fish market in Belém, Pará and from artisanal fishermen in the municipality of Vigia de Nazaré, Pará.

## Material and Methods

**Figure 1:**
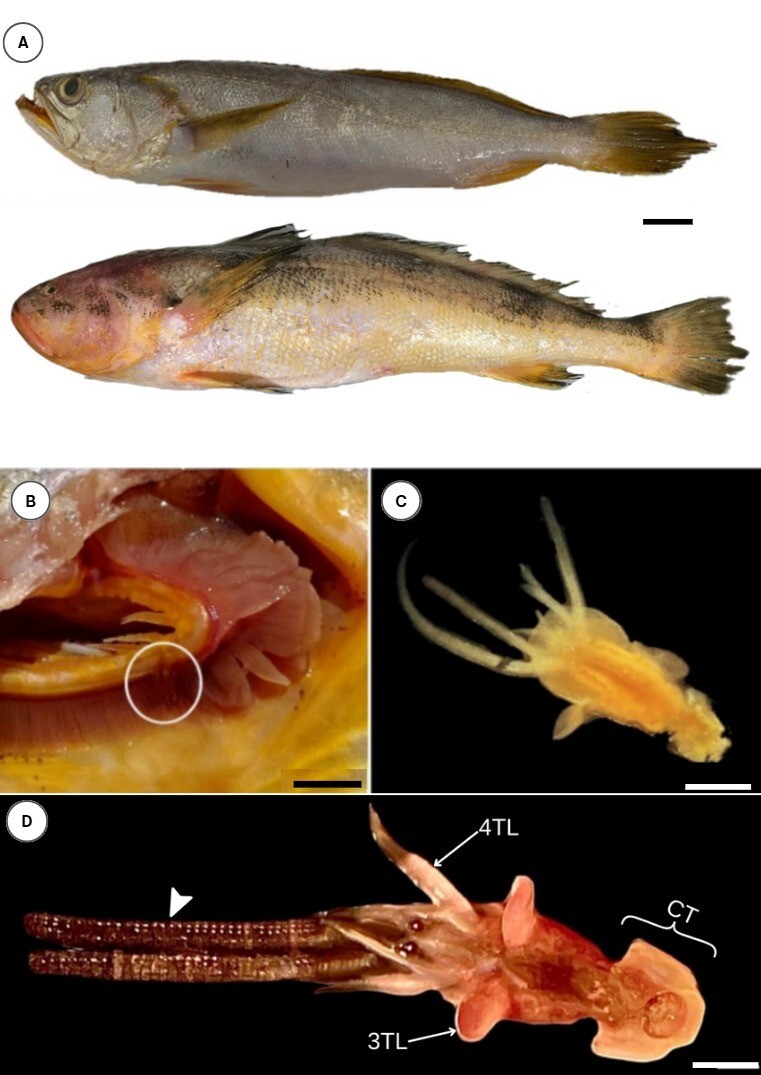
(A) Specimens of *Macrodon ancylodon* (top) and *Nebris microps* (bottom); scale bar: 1 cm; (B) Gills of *Macrodon ancylodon* with the attached copepod (circled); scale bar: 2.5 cm; (C) Photomicrographs of the copepod of genus *Lernanthropus* removed from the gills of *Macrodon ancylodon* under a microscope respectively; scale bar: 1 mm; (D) Photomicrographs of *Lernanthropus* removed from the gills of *Nebris microps* under a stereomicroscope with the attached Cephalothorax - CT, third and fourth Thoracic Leg - 3TL and 4TL and also the Egg Sac (arrow head); scale bar: 0.4 mm.

This study was conducted between December 2020 and November 2022. Four specimens of king weakfish (*M. ancylodon*) (Figure 1A-top) were obtained dead and chilled from fish market in Belém, Pará (1º 28' 28” S; 48º 28' 14.8” W). Additionally, five specimens of smalleye croaker (*N. microps*) (Figure 1A-below) were obtained from the municipality of Vigia de Nazaré (00º 51' 12” S and 48º 08' 41” W) in the Salgado Microregion of northeastern Pará, approximately 100 km from Belém through highways BR-316 and PA-140. The specimens were transported in isothermal containers to the Multidisciplinary Laboratory of Clinical Analysis at the University of Amazonia (UNAMA) and to the Laboratory of Morphomolecular Integration and Technology (LIMT) at the Federal Rural University of Amazonia, both located in Belém, Pará.

The species of fish collected were identified according to Brasil (2016), and examined under a stereomicroscope (loupe) for external assessment, followed by necropsy to analyze internal organs, including gills, gallbladder, and liver. To optimize observations, gills were removed, placed in Petri dishes containing water, and examined individually. Following ectoparasite detection, specimens were carefully removed using forceps, brushes, or by agitating the gills in Petri dishes and visualized with the aid of light microscopy.

Subsequently, small tissue of the infected area (gills) were extracted, compressed between a slide and coverslip, and examined under a light microscope. They were then fixed in an AFA solution (alcohol, formalin and acetic acid) for 48 hours and preserved in 70% ethyl alcohol and 10% glycerin (Eiras et al., 2006).

The specimens of removed copepods were mounted in a Hoyer medium for visualization of sclerotized structures, for taxonomic identification through observation of legs and antennae (Eiras et al., 2006). The parasites were identified morphologically according to Kabata (2003) in light microscope and photographed. The prevalence of parasitism was calculated according to Bush et al. (1997).

## Results and Discussion

### General description

Only one copepod was identified, collected and examined in the gills of each species studied (Figure 1B). It is noteworthy that in addition to the difficulty of finding the parasites of this genus frequently in fish, this result demonstrated that the washing of the gills of the hosts by the fishermen may have influenced the number of identified copepods.

The copepods had an elongated body with a fused anterior cephalic segment and first thoracic segment, forming a cephalothorax slightly wider than their length, wherein this region was narrower than an oval dorsal shield (Figures 1C and 1D). They had five pairs of legs of varying sizes.

### Description of male and female specimens

The male specimen of *M. ancylodon* (Figure 1C) lacked egg-bearing saccular projections. We collected a female specimen of *N. microps* (Figure 1D), with a prominent saccular projection, more developed antennae. It was proportionally larger than the specimen of *M. ancylodon*. It also displayed a slightly more elongated body, suggesting that these may be different species.

### Morphometry of specimens

The specimen found in *M. ancylodon* showed small antennae in the anterior region and apparently firm maxillary hooks (Figure 1C), with a total body length of 4.33 mm and distinct head measuring 0.96 mm in length and 1.15 mm in width.

Additionally, the anterior portion of the trunk (1.28 mm) was narrower than the posterior portion (1.8 mm), with a continuous oval-circular plate extending from the trunk. The uropods were prominent, with a central pair extending beyond the posterior plate, measuring approximately 0.65 mm (Figure 1C). The first and second pairs of legs were underdeveloped, whereas a third pair was lateralized and showed a pyriform shape resembling “fins.” The fourth pair of legs comprised two projections of different lengths, with a fifth pair adjacent to the uropod and slightly shorter than the posterior plate.

**Figure 2:**
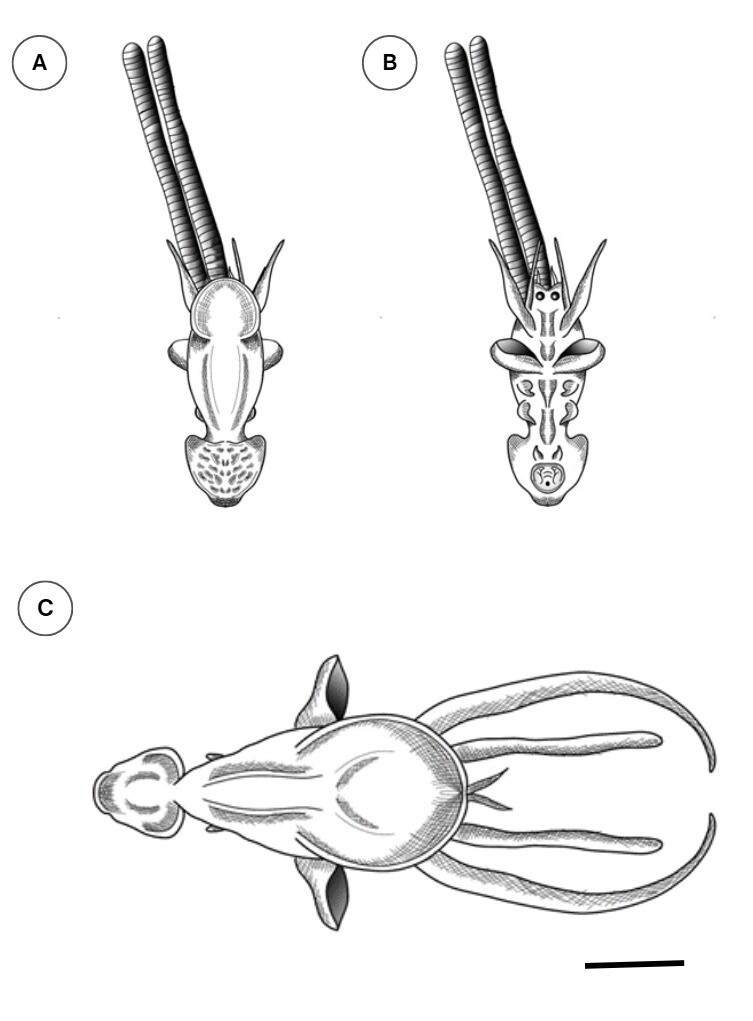
Diagram of (A) dorsal view and (B) ventral view of female *Lernanthropus* sp. specimens found on the gills of *Nebris microps*, and (C) dorsal view of male *Lernanthropus* sp. specimens found on the gills of *Macrodon ancylodon*; scale bar: 0.5 mm.

The anterior portion of the trunk (2.1 mm) was narrower than the posterior portion (3.2 mm), with a continuous oval-circular plate attached to the trunk. The saccular projection measured 4 mm. The remaining morphological features were similar between both sexes (Figure 1C and 1D). The structures of copepods can be observed in the schematic drawing presented in Figure 2.

### Parasitic indices

According to our results, both hosts, king weakfish (*M. ancylodon*) and smalleye croaker (*N. microps*), showed the same prevalence of ectoparasites attached to their gills (25%); however, this may not reflect the actual infection rates in nature, considering the high mobility of these parasites.

Contrasting the present study, Luque & Takemoto (1996) recorded and redescribed *Lernanthropus rathbuni* (Wilson, 1922) from the gill filaments of *Orthopristis ruber* (Cuvier) and *Haemulon steindachneri* (Jordan & Gilbert, 1882) in Sepetiba Bay, Rio de Janeiro, with prevalences of 15.42 and 7.50%, respectively, However, in relation to the attachment area, the parasite's preferred location was the same as in the present study, the gills.

The results of Cavalcanti et al. (2006) were also lower than the results described here, obtained the prevalence for *L. rathbuni*, in the host *Pomadasys corvinaeformis* 17.4% and in relation to the fixation area, the preferred sites by the parasite were gills (97.56%) and the integument (2.44%) of the host. Results superior to the present study were recorded by Čolak et al. (2021), in the host European sea bass *Dicentrarchus labrax* (Linnaeus, 1758), in which the prevalence of *Lernanthropus kroyeri* ranged from 94.4% to 100% and the site of infection was the gills.

According to Henry et al. (2009), local conditions and climate change, such as the increase in sea temperature, can also influence fish stress. And so, hosts with weakened immune systems can result in increased parasite infestation.

In the present study, the proportion of male *Lernanthropus* was dominant in *M. ancylodon* and the proportion of female *Lernanthropus* was dominant in *N. micros*. In studies conducted by Čolak et al. (2021), on the host European sea bass, the proportion of females of *Lernanthropus kroyeri* dominated the population during the entire research period.

The morphological characteristics of these *Lernanthropus* sp. specimens have not yet enabled species identification, and although new sampling efforts have been conducted, no additional specimens have been obtained. This is the first report of these copepods parasitizing these hosts; despite the significance of parasitic copepods in fish, particularly the Lernanthropidae family, only few descriptions of this genus have been published in Brazil, namely, Luque & Paraguassu (2003) on *Lernanthropus leidyi* and *L. caudatus*, which are parasites of teleost fish from Rio de Janeiro.

This is the first record published in parasitism indexed journals by *Lernanthropus* sp. in *M. ancylodon* and *N. microps* in the state of Pará, this being the first report of the occurrence of such parasitism for hosts that are highly appreciated by consumption of the local population and of high commercial importance for the Brazilian Amazon. The importance of visually inspecting fish to detect possible parasites before marketing is highlighted, and products visibly infected by parasites should not be made available on the market.

Studies on the morphological characteristics of parasitic copepods are essential for understanding both the health of national fish and host-parasite relationships.
